# The Impact of Mutations on the Pathogenic and Antigenic Activity of SARS-CoV-2 during the First Wave of the COVID-19 Pandemic: A Comprehensive Immunoinformatics Analysis

**DOI:** 10.3390/vaccines9121410

**Published:** 2021-11-30

**Authors:** Zulqarnain Baloch, Aqsa Ikram, Mohamad S. Hakim, Faryal Mehwish Awan

**Affiliations:** 1 Faculty of Life Science and Technology, Kunming University of Science and Technology, Kunming 650500, China; znbalooch@yahoo.com; 2 Institute of Molecular Biology and Biotechnology (IMBB), The University of Lahore (UOL), Lahore 54000, Pakistan; 3 Department of Microbiology, Faculty of Medicine, Public Health and Nursing, Universitas Gadjah Mada, Yogyakarta 55281, Indonesia; m.s.hakim@ugm.ac.id; 4 Center for Child Health—PRO, Faculty of Medicine, Public Health and Nursing, Universitas Gadjah Mada, Yogyakarta 55281, Indonesia; 5 Department of Medical Lab Technology, The University of Haripur (UOH), Haripur 22620, Pakistan; faryal.mehwish@uoh.edu.pk

**Keywords:** mutation, RdRp, spike, SARS-CoV-2, 3CLpro, epitope

## Abstract

An in-depth analysis of first-wave SARS-CoV-2 genome is required to identify various mutations that significantly affect viral fitness. In the present study, we performed a comprehensive in silico mutational analysis of 3C-like protease (3CLpro), RNA-dependent RNA polymerase (RdRp), and spike (S) proteins with the aim of gaining important insights into first-wave virus mutations and their functional and structural impact on SARS-CoV-2 proteins. Our integrated analysis gathered 6000 SARS-CoV-2 sequences and identified 92 mutations in S, 37 in RdRp, and 11 in 3CLpro regions. The impact of these mutations was also investigated using various in silico approaches. Among these, 32 mutations in S, 15 in RdRp, and 3 in 3CLpro proteins were found to be deleterious in nature and could alter the structural and functional behavior of the encoded proteins. The D614G mutation in spike and the P323Lmutation in RdRp are the globally dominant variants with a high frequency. Most of the identified mutations were also found in the binding moiety of the viral proteins which determine their critical involvement in host–pathogen interactions and may represent drug targets. Furthermore, potential CD4^+^ and CD8^+^ T cell epitopes were predicted, and their overlap with genetic variations was explored. This study also highlights several hot spots in which HLA and drug selective pressure overlap. The findings of the current study may allow a better understanding of COVID-19 diagnostics, vaccines, and therapeutics.

## 1. Introduction

Severe acute respiratory syndrome coronavirus 2 (SARS-CoV-2), one of the seven known human-infecting coronaviruses, is a highly transmissible and pathogenic virus [1]. It belongs to the *Betacoronavirus* genus and is an enveloped, positive-sense, single-stranded RNA virus [2]. RNA viruses exhibit high mutation rates due to the low fidelity displayed by their RNA-dependent RNA polymerase (RdRp) [3]. Mutations can be beneficial for virus’s survival as they can render them more pathogenic, facilitate immune escape, and contribute to drug resistance [4]. Furthermore, mutations may lead to different phenotypic changes in viruses. If a mutation changes an amino acid, it may also change the stability, functionality, and antigenicity of the related protein. In this context, if the mutation is present in a structurally important part of the protein, normal functions may be lost and have a lethal effect. As SARS-CoV-2 spreads around the globe, it is mutating and acquiring genetic changes. Similarly, continuous changes in genetics and antigenicity of influenza viruses significantly affect vaccine efficacy. This evolving nature of viruses through gradual accumulation of mutations requires a constant updating of vaccine strains in order to make sure that the vaccines have similar or identical antigenic profiles to those of the circulating strains and are effective in controlling the disease [5,6]. A comparison of the similarities and differences between coronaviruses and influenza viruses may assist us in understanding how those similarities and differences could impact potential COVID-19 vaccines. Similarly, RNA viruses exploit all known mechanisms of genetic variation to guarantee their survival. The strategies adopted by single-stranded RNA viruses such as influenza or HIV are not used by SARS-CoV-2. In contrast to all other known RNA viruses, coronaviruses do not mutate very rapidly. However, few mutations, such as D614G in spike (S) and p323L in RdRp, have been rapidly evolving in SARS-CoV-2 genome. Among these, the D614G mutation is responsible for increased transmissibility [7]. Thus, the beneficial effect of mutations in SARS-CoV-2 cannot be avoided. Hence, they have a great impact on human health, which suggests that any new mutations in SARS-CoV-2 can be hazardous during this rapidly escalating outbreak. Studies performed over the past few months have revealed that SARS-CoV-2 has acquired some evolving mutations in its human host [1,8].

The functional and structural consequences of these mutations are unknown, and it will be substantial to determine their impact on virus transmissibility and pathogenicity in humans. The analysis of genetic sequence data freely available in NCBI (https://www.ncbi.nlm.nih.gov/nuccore, accessed on 15 November 2021) and Global Initiative on Sharing All Influenza Data (GISAID; https://www.epicov.org, accessed on 15 June 2021) can shed light on key epidemiological parameters of SARS-CoV-2, including evolving mutations. Therefore, we kept our focus on SARS-CoV-2 mutations lying within RdRp, 3C-like protease (3CLpro), and S proteins in an attempt to assess the spread of new viral variants across the countries and also the real functional and structural impact of these mutations on the pathogenicity and antigenicity of SARS-CoV-2. These viral proteins are considered among the primary targets for vaccine and antiviral drug development [9].

A more comprehensive understanding of virus mutations, their evolution, and their overall effect on immunogenicity can be achieved by a genomic analysis of sequence data that can further guide various experimental studies. The availability of such comprehensive data is enabling researchers to use various bioinformatics tools in an attempt to extract useful hidden clinical and molecular information [10]. There is a need to uncover deleterious mutations and their pathogenic variants using the readily available data and to further explore their impact at the molecular level. In silico tools can be effectively utilized for prioritizing different variations in a cost-efficient manner and to further investigate the structural, functional, and immunogenic consequences of specific mutations [11]. In this study, all available genomic information regarding the first wave of SARS-CoV-2 was retrieved, and various in silico approaches were used to provide an insight into the pathogenic and immunogenic landscape of various mutations in selected viral proteins.

The main aim of the study was to understand and predict various pathogenic variants of first-wave SARS-CoV-2 RdRp, 3CLpro, and S proteins. Overall, 32 mutations in S, 15 in RdRp, and 3 in 3CLpro were predicted in this study, which are involved in major phenotypic damage and could alter the structural and functional behavior of the encoded proteins. To further understand the viral immune escape strategies, we examined the overlap between the reported mutations and immune-driven mutations in SARS-CoV-2 genes. The current study also unveiled a significant co-occurrence of these mutations and T cell epitope mutations that may affect both therapeutic and the host immune responses.

## 2. Materials and Methods

### 2.1. Sequence Retrieval

Complete genome sequences of first-wave SARS-CoV-2 (*n* = 6000) were downloaded from GenBank and GISAID until 15 June 2021. Genome sequence NC_045512 was used as a reference sequence and is considered a wild-type (WT) sequence. From these complete genome sequences, sequences of S, RdRp, and 3CLpro regions were screened out.

### 2.2. Sequence Alignment and Mutation Analysis

Protein sequences of S, RdRp, and 3CLpro regions were first aligned with the reference sequence (NC_045512) using CLC workbench 7 and Bioedit [12]. The origin and position of each mutation within these viral proteins were assessed.

### 2.3. The Impact of Mutations on the Structural and Functional Properties of the Encoded Viral Proteins

The prediction of different mutations that alter the structure and functions of SARS-CoV-2 proteins can actually guide the design of pharmaceutical compounds and initiate vaccine design and development. Thus, to estimate the effect of the identified mutations on various structural and functional features of SARS-CoV-2 viral proteins, the following analyses were performed.

#### 2.3.1. Predicting the Functional Impact of Mutations

To characterize mutations as neutral or deleterious to the structure and function of the encoded proteins, SIFT [13], PhD-SNP [14], and SNAP2 tools [15] were employed. SIFT predicts the functional importance of an amino acid variations based on the conservation and alignment of highly similar orthologous and paralogous protein sequences. Substitutions with probability score less than 0.05 are considered deleterious, while values ≥0.05 are considered to be tolerated, i.e., they may have no significant effect.

PhD-SNP is a support vector machine-based software and predicts whether a nucleotide substitution may cause a disease or may remain neutral. The SNAP2 (screening for non-acceptable polymorphisms) program (www.rostlab.org/services/SNAP/, access on 15 June 2021) makes predictions regarding the functionality of variant proteins.

#### 2.3.2. Predicting Protein Stability Changes upon Mutations

The prediction of mutations’ impact on the conformation, flexibility, and stability of proteins is also required to gain insights into the structure–function relationships of the encoded proteins. Protein stability is the basic characteristic that affects the function, activity, and regulation of proteins [16]. Free energy related to protein unfolding is a key index of protein stability. Therefore, by analyzing the influence of a mutation on free energy, its effect on protein stability can be accurately determined. To quantitatively predict changes in protein conformation, flexibility, and stability due to mutations, i-Mutant version 2.0 [17], DUET [18], and Dynamut [16] web servers were used. For DUET and Dynamut prediction, the 3D structures of RdRp and S were predicted using i-TASSER, while the crystal structure (5re5) of 3CLpro was retrieved from protein data bank (PDB).

#### 2.3.3. Mutation Screening

In order to recapitulate the predictive results of the above-mentioned tools, a scoring criterion was set (0–6). If a mutation was predicted to be “harmless” or “neutral” by all tools, it would score 0; in contrast, it would get a score if any of the tools predicted it as a “harmful” or “pathogenic” mutation on the basis of the number of tools predicting it. Mutations predicted by four or more tools (thus, with a score ≥4) were then screened for further evaluations.

#### 2.3.4. Normal Mode Analysis

Normal mode analysis was performed via the iMod server (iMODS) (http://imods.chaconlab.org, accessed on 15 June 2021) by using the basic default values for all the parameters mentioned. Only highly pathogenic mutations (with a score ≥4) were considered for this analysis.

#### 2.3.5. Mapping the Ligand Binding Sites with Mutations

To find the location of the screened mutations within the drug binding sites of viral proteins, the COACH (http://zhanglab.umich.edu/COACH/, accessed on 15 June 2021) and CASTP (http://sts.bioe.uic.edu/castp/index.html?2r7g, access on 15 June 2021) servers were used. These servers predict protein–ligand binding sites; thus, these sites were evaluated for the presence of any pathogenic mutations. Mutations lying within these regions were then screened to determine the negative effects on the targeted proteins and their possible interactions.

#### 2.3.6. Epitope Mapping

To understand the strategies of viral immune escape, we examined the potential overlap between the reported mutations and immune-driven mutations in the S protein. MHC class I- and II-restricted T cell epitopes from the consensus sequence of first-wave SARS-CoV-2 (*n* = 6000) were predicted using the online epitope prediction software HLA–peptide binding predictor HLAPred (www.imtech.res.in/raghava/hlapred/, accessed on 15 June 2021). Epitopes binding to the highest number of alleles were selected for further analysis. For the prediction of B cell epitopes, the Immune Epitope Database (IEDB) [19] was utilized.

#### 2.3.7. Co-Occurring Mutations in Reported and Predicted Epitopes

A comprehensive analysis was carried out to identify any potential overlap between the reported mutations and epitope mutations. This overlap was defined as multiple T and B cell epitopes that also incorporate the reported mutations. The effect of these mutations on overall antigenicity was calculated by using the Vaxijen server [20].

## 3. Results

### 3.1. Mutations Residing in S, RdRp, and 3CLpro Sequences

**Figure 1:**
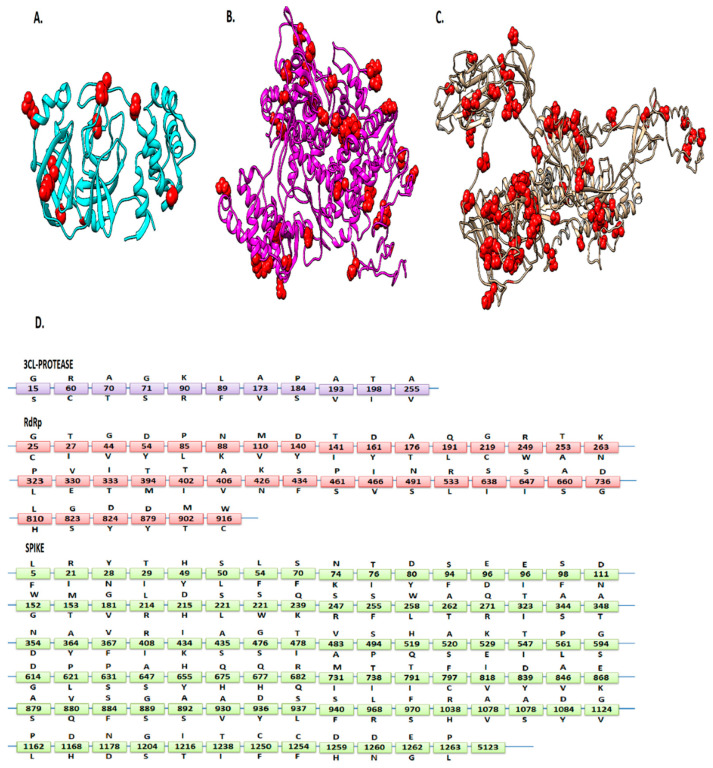
Mutation representation: The locations of 3CLpro (**A**), RdRp (**B**), and S (**C**) of SARS-CoV-2 mutations are presented in red spheres. (**D**) The letters above the boxes refer to the wild-type amino acid, and the letters below the boxes are relevant substitutions reported in this study.

Alignment of 6000 first-wave SARS-CoV-2 protein sequences with the reference sequence Wuhan-Hu-1 (Accession NC_045512) revealed 92 mutations in S, 37 in RdRp, and 11 in 3CLpro regions (Table 1 and Figure 1). These mutations were found in many countries, including the USA, China, Australia, South Korea, India, Peru, Sweden, Spain, Vietnam, England, Pakistan, Turkey, Germany, France, Greece, Sri Lanka, South Africa, Colombia, Iran, and Malaysia. This indicates that the virus has a significantly high evolution rate in various geographical regions which increases viral fitness. D614G (50%) and P323L (49%) mutations showed the highest frequency among the screened sequences. Moreover, the mutation frequencies of P323L (49%) and D614G (50%) were found to be similar within the period from 15 January 2020 to 15 July 2021.

To further evaluate the effect of the given mutations on the structure and function of the respective proteins, a variety of in silico SNP prediction algorithms were used. NC_045512 was considered the wild-type genome. Its S and RdRp structures were predicted by i-TASSER, whereas the crystal structure of SARS-CoV-2 3CLpro was retrieved from PDB (PDB ID: 5re5).

### 3.2. Analyzing the Effect of Mutations on Structural and Functional Stability of the Respective Proteins

Six pathogenicity prediction software tools, including SIFT [13], PhDSNP [14], SNAP2 [15], I-Mutant version 2.0 [17], DUET [18], and Dynamut [16], were employed to predict the effects of a total of 140 mutations in S (92), RdRp (37), and 3CLpro (11). According to SIFT analysis, in the S protein, 34 mutations were found to be deleterious, and 58 mutations appeared to be tolerated (neutral) in nature. In the RdRp protein, 20 mutations were declared non-tolerated, while 17 were tolerated. In the 3CLpro protein, three mutations were predicted as non-tolerated, and seven mutations were tolerated.

**Figure 2:**
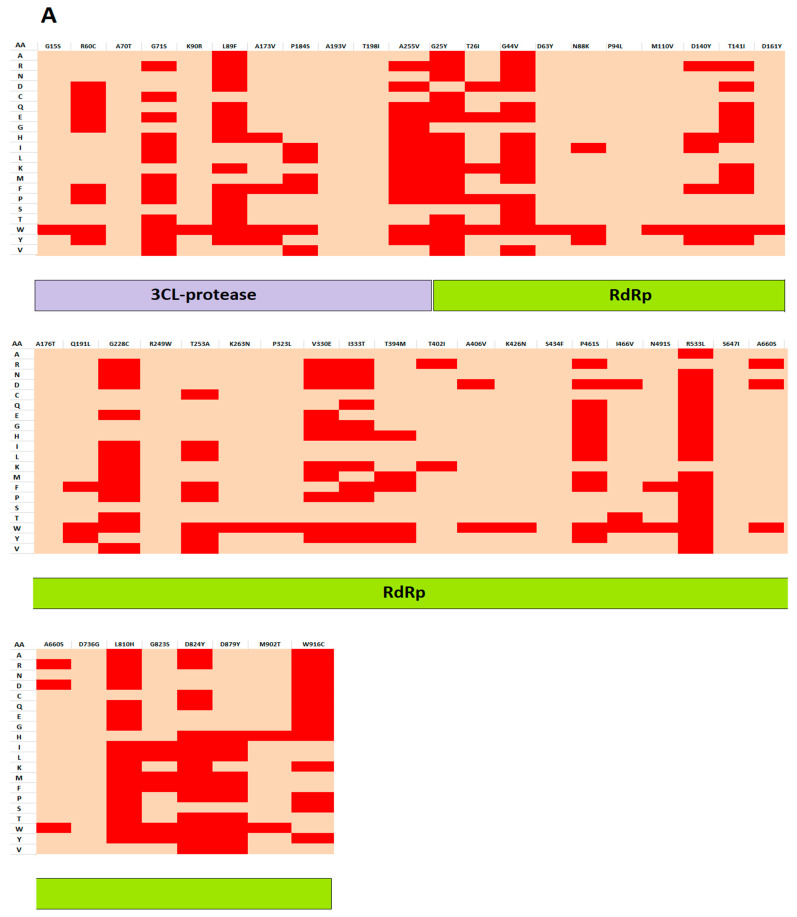

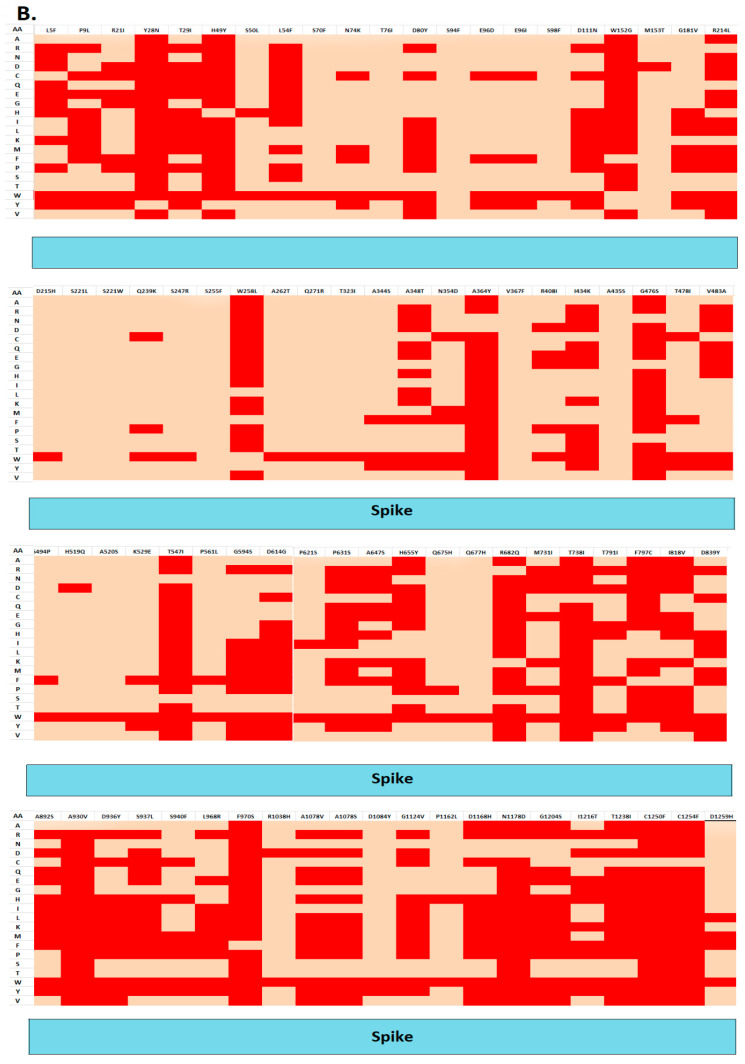
Heatmap representation of pathogenic and non-pathogenic mutations in 3CLpro, RdRp (**A**), and S proteins (**B**). Heatmap representation showing possible substitutions at each pathogenic-mutation position of a protein. Dark red indicates a high score (strong signal for an effect), and green indicates a low score (strong signal for a neutral/no effect) based on SNAP2 analysis. The *y*-axis reports the amino acids, and the top *x*-axis reports the mutations.

PhD-SNP predicted 20 mutations in the S protein as damaging or deleterious, 11 in RdRp, and two in 3CLpro protein. SNAP2 revealed that 29 mutations in S, 10 in RdRp, and 3 in 3CLpro could affect the overall function of these viral proteins. It also predicted the type of amino acid that affects the function of the related protein when altered at a particular position. Based on this prediction analysis, a heat map was generated depicting the ability of the identified amino acids to change the function of the respective viral proteins (Figure 2A,B).

**Figure 3:**
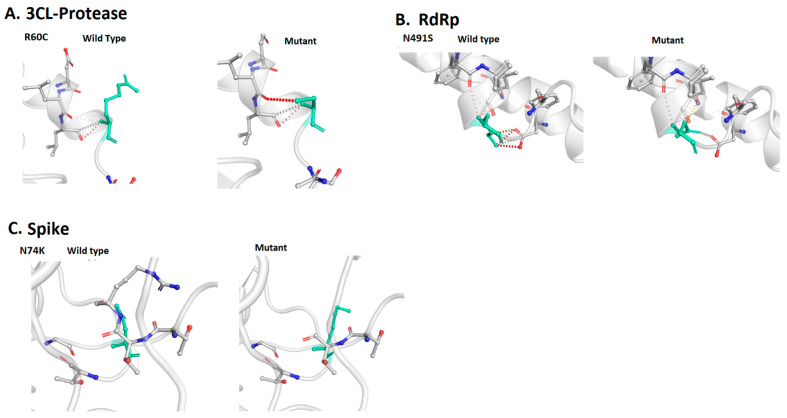
The effects of mutations (R60C, 3CLpro; N491S, RdRp; and N74K, S) on the structural stability of viral proteins predicted by the Dynamut web server.

Findings of i-Mutant showed that out of 92 mutations, 71 are deleterious for the S structure. They also revealed that 32 mutations in RdRp and 7 in 3CLpro are deleterious mutations. According to DUET, 68 mutations in S, 23 mutations in RdRp, and 8 mutations in 3CLpro proteins are deleterious in nature. Findings of Dynamut suggested that 65 mutations in S, 25 in RdRp, and 8 in 3CLpro can affect the structural conformation of the respective viral proteins. They also predicted interatomic interactions of wild-type and mutant amino acids with the environment based on atom type, interatomic distance, and angle constraints. Some of the selected deleterious mutations of S, RdRp, and 3CLpro, as well as an interatomic interaction analysis, are shown in Figure 3.

Details of all predicted mutations and their possible effects on the encoded proteins are reported in Table 1. These analyses predicted mutations that could affect the structural stability of proteins by changing their flexibility and rigidity. To evaluate these mutations, six tools were employed, each using different strategies and parameters to predict deleterious mutations. The mutations with more positive results were more likely to be truly deleterious. Mutations observed to be deleterious by more than three prediction algorithms were classified as high-risk (see Material and Methods).

**Figure 4:**
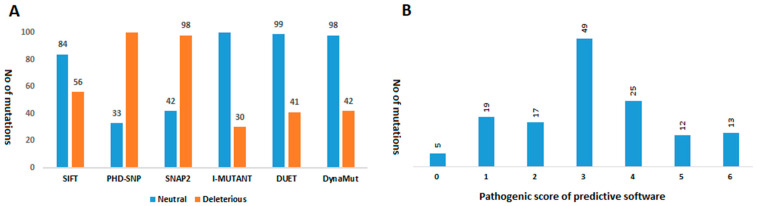
Prediction of pathogenicity of nsSNPs by SIFT, PhD-SNP, SNAP2.0, I-MUTANT, DUET, and DynaMut software. (**A**) Number of “pathogenic” or “neutral” protein variants predicted by each bioinformatics tool. (**B**) Number of protein variants with different pathogenicity scores predicted by the six bioinformatics tools.

Figure 4 shows the prediction results of six computational tools. We found that five mutations were predicted to be neutral with a score of 0, while 19, 17, 49, 25, 12, and 13 mutations obtained a score of 1, 2, 3, 4, 5, and 6, respectively (Figure 4). Based on the given criteria, 32 mutations in S, 15 in RdRp, and 3 in 3CLpro (Table 1) met these criteria (score ≥ 4) and were chosen for further analysis (Figure 4). Among these pathogenic mutations, D614G (score = 4) in the S region has already been reported to be associated with greater infectivity [7]. Another highly prevalent mutation (P323L) in the RdRp region was found to be neutral (score = 2), whereas its infectivity has not been reported so far. Finally, all deleterious mutations were mapped on the 3D structure of the viral proteins. It was observed that all these mutations were uniformly distributed on the viral protein structures.

### 3.3. Localization of the Deleterious Mutations within the Binding Sites of Viral Proteins

The 3D structure of the SARS-CoV-2 protease was retrieved from PDB with PDB ID 5RE5. For S and RdRp proteins, top i-TASSER-predicted models were selected on the basis of the C-score. The RAMPAGE and ProSA web servers were further used to verify the reliability of the predicted models.

The results of the predicted 3D RdRp model showed 83% of the residues in the favored region, 10.8% in the additional allowed region, and 6.2% in the outlier region. The tertiary structure of the S protein showed 75.2% of the residues in the favored region, 14.8% in the allowed regions, and 10% in the outlier regions, strongly indicating a good stereo-chemical quality of the predicted structures. By using these 3D structures, the COACH and CASTP servers predicted the possible ligand-binding sites of these proteins. The ligand-binding sites predicted by both servers were considered as potential binding sites. It was observed that in the S protein, 22 out of 37 deleterious mutation positions, including 28, 71, 74, 96, 152, 348, 435, 675, 682, 797, 824, 846, 860, 930, 936, 970, 1168, 1178, 1168, 1250, 1258, and 1259, lie in the ligand binding site. In RdRp, 13 predicted deleterious mutation positions (25, 44, 63, 110, 228, 249, 333, 426, 491, 660, 810, 824, and 916) lie in the ligand-binding sites, while in 3CLpro, all selected deleterious mutation positions (15, 60, and 89) lie within the binding site.

### 3.4. Normal Mode Analysis of Highly Deleterious Mutations

iMODs is a user-friendly interface for normal mode analysis. It provides detailed information about mobility (B-factors), eigenvalues, covariance map, and deformability of a protein. The eigenvalue represents the total mean square fluctuations and is related to the energy required to deform a structure. The lower eigenvalues represent the easier deformation of a protein. iMODs analysis revealed that all selected deleterious mutations decrease the eigenvalues of RdRp, S, and 3CLpro proteins, indicating the deleterious effects of the evolving mutations in the selected viral proteins (Figure S1).

### 3.5. Overlap of the Reported Mutations within the Predicted Epitopes

Only those epitopes that were shown to bind the highest number of alleles overlapped with B cell epitopes and were involved in SARS-CoV-2 protection or clearance (HLA-B*15:03) [7] were screened out. Several HLA-restricted and B cell epitopes were found to perfectly overlapped with or be flanked by mutations. However, a single mutation may occur within more than one HLA-restricted epitope. This finding suggests the existence of mutations and immune-driven variations at a single site.

### 3.6. Estimating the Antigenicity of Epitopes

SARS-CoV-2 may modify its epitopes so that they are not recognized by T cells, and this ultimately leads to immune escape. The online tool Vaxijen [20] was employed to find the effect of each mutation on the antigenicity of the epitopes (Table 2). Interestingly, in many cases, the mutations reduced the antigenicity of epitopes (T1–T32) compared to the wild-type sequences (T1–T32). It was observed that the antigenicity of the epitope with the deleterious mutation D614G decreased. Some epitopes with mutations maintained their antigenicity, while others showed increased antigenicity (Table 2). This suggests that few mutations within epitopes have reduced antigenicity, thus decreasing the effective role of T cells.

## 4. Discussion

What we know about single-stranded RNA viruses is not true for coronaviruses. In contrast to all other known RNA viruses, coronaviruses do not mutate as much. However, over 10,000 single-nucleotide polymorphisms (SNP) in many subtypes of SARS-CoV-2 have been observed [21]. This indicates that the evolution of SARS-CoV-2 is characterized by the emergence of sets of mutations that impact virus transmissibility and antigenicity. The current study was based on in silico mutagenesis analysis of first-wave SARS-CoV-2 RdRp, S, and 3CLpro proteins with the aim to identify mutations and their possible structural and functional impact on the encoded viral proteins. In this study, 92 mutations in S, 37 in RdRp, and 11 in 3CLpro proteins were identified in the sequence data reported by various countries. The effect of such mutations on the structure and function of the respective viral proteins is important to predict the evolutionary potential of the viral proteins. However, in silico prediction of the impact of amino acid variants on proteins’ structure and function may, sometimes, be considered as an alternative to or a pre-study indicator of in vitro expression level studies [22]. In addition, the interpretation of the proteomic variants in light of their phenotypic effects is one of the emerging crucial tasks we have to perform in order to advance our understanding of how these variants affect SARS-CoV-2 proteins structural and functional behavior. The proteins RdRp, S, and 3CLpro of SARSCoV-2 are important targets for antiviral drug and vaccine development [23] and, thus, were selected for bioinformatics analysis in this study. Any mutation in these viral proteins could be either beneficial or pathogenic (deleterious) for the virus [3]. Therefore, we identified mutations in the selected viral proteins as well as the possible impact of these mutations on the overall structure, function, and immunogenicity of these proteins.

It was observed that most of the mutations lie in the S region (97), followed by RdRp (37), and 3CLpro (11). A highly mutated amino acid was observed at the position D614G (50%) in the S protein and P323L (49%) in the RdRp protein. By using various in silico algorithms and selected scoring criteria (0–6), it was estimated that 32 mutations in S, 15 in RdRp, and 3 in 3CLpro proteins were deleterious in nature and probably affect the overall structure and function of these viral proteins. Among these mutations, D614G is highly prevalent and associated with greater infectivity of SARS-CoV-2. It was also found to be pathogenic in nature (score = 4), thus validating our results. Another highly prevalent mutation, P323L in RdRp, was found to be neutral (score = 2). Similarly, the remaining mutations are rare and do not appear to be more deleterious.

In support of this, few studies have also revealed that variations in certain epitopes can critically influence the outcome of immune responses and antiviral treatments in patients infected by SARS-CoV-2. The S protein facilitates the attachment of the virus to host cell surface receptors and is a major target for neutralizing antibodies [24]. Therefore, mutations that change the overall antigenicity of the S protein are of great importance. The present study was also designed to evaluate the co-occurrence of viral mutations with T cell (CD4+ and CD8+) and B cell epitope mutations. The analysis of these epitopes showed that mutations were frequent within the predicted epitopes compared to the regions outside of these epitopes. We observed that this overlap either decreased, sustained, or enhanced the antigenicity of epitopes (Table S1).

## 5. Conclusions

Together, these findings have implications for our understanding of SARS-CoV-2 mutations. These mutations not only affect the structural and functional abilities of viral proteins, but also might affect the binding affinities of these viral proteins with various drugs, as most of these pathogenic mutations are also present in ligand-binding regions. This characterization of drug and vaccine target protein variants of SARS-CoV-2 could help us understand the pathogenesis, treatment options, vaccines design, and diagnostic strategies of COVID-19. It would potentially be significant to characterize the impact of these identified pathogenic mutations by employing various in vitro and molecular approaches.

## Figures and Tables

**Table 1:** Prediction of pathogenic mutations: Variations in 3CLpro (A), RdRp (B), and S (C) of SARS-CoV-2 that were predicted to be “deleterious” and “pathogenic” by all the six pieces of software.

(**A**)								
	**Mutations**	**SIFT**	**PHD-SNP**	**SNAP2**	**i-Mutant**	**DUET**	**DynaMut**	**SCORE**
3CL-protease	G15S	-	-	√	√	√	√	4
	R60C	√	√	√	√	√	√	6
	A70T	-	-	-	√	√	√	3
	G71S	-	-	-	√	√	√	3
	K90R	-	-	-	√	√	√	3
	L89F	√	√	√	√	√	√	6
	A173V	-	-	-	-	-	-	0
	P184S	-	-	-	√	√	√	3
	A193V	-	-	-	-	-	-	0
	T198I	-	-	-	-	-	-	0
	A255V	-	-	-	√	√	√	3
(**B**)								
RdRp	G25Y	√	√	√	√	√	√	6
	T26I	-	-	-	√	√	√	3
	G44V	-	-	√	√	√	√	4
	D63Y	√	-	-	√	√	√	4
	N88K	-	-	-	√	√	√	3
	P94L	-	-	-	√	√	√	3
	M110V	√	-	-	√	√	√	4
	D140Y	√	√	-	-	-	√	3
	T141I	√	-	-	√	-	-	2
	D161Y	-	√	-	-	-	-	1
	A176T	-	-	-	√	√	√	3
	Q191L	√	-	-	√	-	-	2
	G228C	√	√	-	√	√	√	5
	R249W	√	-	√	√	√	√	5
	T262A	-	-	-	√	-	-	1
	K263N	-	-	-	-	√	√	2
	P323L	-	-	√	√	-	-	2
	V330E	√	√	√	√	√	√	6
	I333T	√	√	-	√	√	√	5
	T394M	-	-	√	√	-	-	2
	T402I	√	-	-	√	-	-	2
	A406V	-	-	-	√	√	√	3
	K426N	√	-	-	√	√	√	4
	S434F	√	-	-	-	√	√	3
	P461S	-	-	-	√	√	√	3
	I466V	-	√		√	√	√	4
	N491S	√	-	-	√	√	√	4
	R533L	√	√	-	√	-	-	3
	S647I	√	-	-	-	-	-	1
	A660S	√	-	-	√	√	√	4
	D736G	-	-	-	√	-	-	1
	L810H	√	√	√	√	√	√	6
	G823S		-		√	√	√	3
	D824Y	√	√	√	√	√	√	6
	D879Y	-	-	√	√	-	-	2
	M902T	-	-	√	√	-	-	2
	W916C	√	√	-	√	√	√	5
(**C**)								
Spike	L5F	-	-	-	√	√	√	3
	P9L	-	-	√	√	√		3
	R21I	-	-	-	√	-	-	1
	Y28N	-	-	√	√	√	√	4
	T29I	√	-	-	-	-	-	1
	H49Y	√	-	-	-	-	-	1
	S50L	-	-	-	√	-	-	1
	L54F	-	-	-	√	√	√	3
	S71F	√	√	-	√	√	√	5
	N74K	√	√	√	√	√	√	6
	T76I	-	-	-	√	-	-	1
	D80Y	-	-	√	√	-	-	2
	S94F	√	-	-	-	√	√	3
	E96D	√	-	-	√	√	√	4
	E96I	√	-	-	√	√	√	4
	S98F	-	-	-	√	√	√	3
	D111N	-	-	-	√	√	√	3
	W152G	-	-	√	√	√	√	4
	M153T	-	-	-	√	√	√	3
	G181V	-	-	-	√	√	√	3
	R214L	-	-	√	√	-	-	2
	D215H	-	-	-	√	-	-	1
	S221L	-	-	-	-	√	-	1
	S221W	√	-	-	-	-	-	1
	Q239K	-	-	-	-	√	√	2
	S247R	√	-	-	-	-	-	1
	S255F	-	-	-	-	√	√	2
	W258L	-	-	-	√	√	√	3
	A262T	-	-	-	√	√	√	3
	Q271R	-	-	-	√	√	√	3
	T323I	-	-	-	√	-		1
	A344S	-	-	-	√	√	√	3
	A348T	√	-	-	√	√	√	4
	N354D	-	-	-	√	√	√	3
	D364Y	√	-	-	√	-	-	1
	V367F	-	-	-	√	√	√	3
	R408I	-	-	-	√	-	-	1
	I434K	-	-	√	√	√	√	4
	A435S	√	-	-	√	√	√	4
	G476S	-	-	-	√	√	-	2
	T478I	-	-	-	√	√	√	3
	V483A	-	-	-	√	√	√	3
	S494P	-	-	-	-	√	√	2
	H519Q	-	-	-	-	-	-	0
	A520S	-	-	-	-	-	-	0
	K529E	-	-	-	√	-	-	1
	T547I	-	-	√	√	-	-	2
	P561L	-	-	-	√	-	-	1
	G594S	-	-	-	√	√	√	3
	D614G	-	-	√	√	√	√	3
	P621S	-	-	-	√	√	√	3
	P631S	-	-	-	√	√	√	3
	A647S	-	-	-	√	√	√	3
	H655Y	√	√	√	-	-	-	3
	Q675H	-	-	√	√	√	√	4
	Q677H	-	-	-	√	√	√	3
	R682Q	-	-	√	√	√	√	4
	M731I	-	-	-	√	√	√	3
	T739I	√	√	√	√	-	-	4
	T791I	-	-	-	√	-	-	1
	F797C	√	√	√	√	√	√	6
	I818V	-	-	-	√	√	√	3
	D839Y	√	√	√	-	√	√	5
	A846V	-	-	√	√	√	√	4
	V860Q	√	√	√	√	√	√	6
	E868K	-	-	-	√	√	√	3
	A879S	-	-	-	√	√	√	3
	S884F	√	√	√	-	√	√	5
	G889S	√	-	-	√	√	√	4
	A892S	-	-	-	√	√	√	3
	A930V	√	√	√	√	√	√	6
	D936Y	√	√	√	√	√	√	6
	S937L	√	-	√	-	√	√	4
	S940F	√	√	√	-	√	√	5
	L966R	√	√	√	-	√	√	5
	F970S	√	√	√	√	√	√	6
	A1078V	√	√	√	-	-	-	3
	A1078S	-	-	-	-	√	√	2
	D1084Y	-	√	√	-	√	√	4
	G1124V	-	-	-	√	√	√	3
	P1162L	-	√	-	√	√	√	4
	D1168H	√	√	√	√	√	√	6
	N1178D	√	-	-	√	√	√	4
	G1204S	-	-	-	√	√	√	3
	I1216T	√	√	-	√	√	√	5
	T1238I	√	-	√	-	-	-	2
	C1250F	√	√	√	√	√	-	5
	C1254F	√	√	√	√	-	√	5
	D1259H	√	-	-	√	√	√	4
	D1260N	-	-	-	√	√	√	3
	E1262G	-	-	-	√	√	√	3
	P1263L	√	-	-	√	√	√	4

**Table 2:** Decreased epitope binding potential due to the presence of mutations: The effect of reported mutations on the antigenicity (threshold level = 0.5) of predicted epitopes.

Protein	Epitope Position	Mutation Position	Name	Predicted Epitopes	Antigenicity (without Mutations)	Predicted Epitopes with Mutations	Antigenicity (with Mutations)
**Spike MHCI**	69	S71F	T1	HVSGTNGTK	1	HVS/FGTNGTK	0.6
	515	H519Q	T2	FELLHAPAT	0.5	FELLH/QAPAT	0.1
	515	A520S	T3	FELLHAPAT	0.5	FELLHA/SPAT	0.2
	545	T547I	T4	GLTGTGVLT	1	GLT/IGTGVLT	0.8
	612	D614G	T5	YQDVNCTEV	1.6	YQD/GVNCTEV	1.3
	654	H655Y	T6	EHVNNSYEC	1	EH/YVNNSYEC	0.9
	1210	I1216T	T7	IKWPWYIWL	0.9	IKWPWYI/TWL	0.6
	1257	E1262G	T8	DEDDSEPVL	0.5	DEDDSE/GPVL	0.33
**Spike MHCII**	231	Q239K	T9	IGINITRFQ	1.33	IGINITRFQ/K	1.2
	318	T323I	T10	FRVQPTESI	0.9	FRVQPT/IESI	1
	353	N354D	T11	WNRKRISNC	0.5	WN/DRKRISNC	0.4
	512	H519Q	T12	VLSFELLHA	1	VLSFELLH/QA	0.77
	512	A520S	T13	VLSFELLHA	1	VLSFELLHA/S	0.8
**3CL-protease MHCI**	68	A70T	T14	VQAGNVQLR	1.9	VQA/TGNVQLR	1.8
	68	G71S	T15	VQAGNVQLR	1.9	VQAG/SNVQLR	1.4
**3CL-protease MHCII**	57	R60C	T16	LLIRKSNHN	0.7	LLIR/CKSNHN	0.3
	67	G71S	T17	FLVQAGNVQ	0.8	FLVQAG/SNVQ	0.7
**RdRp** **MHCI**	18, 24	G25Y	T18	RLTPCGTGT	1.1	RLTPCGTG/YT	0.6
				TGTSTDVVY		TG/YTSTDVVY	
	18, 24	T26I	T19	RLTPCGTGT	1.1	RLTPCGTGT/I	0.9
				TGTSTDVVY	0.7	TGT/ISTDVVY	0.3
	37	G44V	T20	IYNDKVAGF	0.5	IYNDKVAG/VF	0.1
	90	P94L	T21	LKDCPAVAK	0.6	LKDCP/LAVAK	0.5
	155	D161Y	T22	DYFNKKDWY	1.2	DYFNKKD/YWY	0.3
	174	A176T	T23	VYANLGERV	0.8	VYA/TNLGERV	0.1
	184	Q191L	T24	QALLKTVQF	0.5	QALLKTVQ/LF	0.2
	400	T402I	T25	ALTNNVAFQ	1.2	ALT/INNVAFQ	0.4
	429	S434F	T26	FKEGSSVEL	0.6	FKEGS/FSVEL	0.2
	527	R533L	T27	LFAYTKRNV	1	LFAYTKR/LNV	0.9
	897	M902T	T28	GHMLDMYSV	0.4	GHMLDM/TYSV	0.1
**RdRpMHCII**	37	G44V	T29	IYNDKVAGF	0.5	IYNDKVAG/VF	0.1
	241	R249W	T30	LMPILTLTR	0.9	LMPILTLTR/W	1.1
	387	T394M	T31	LLLDKRTTC	1.33	LLLDKRTT/MC	1

## Data Availability

Not applicable.

## Supplementary Materials

The following are available online at https://www.mdpi.com/article/10.3390/vaccines9121410/s1, Figure S1: Normal mode analysis of WT (A) and mutant 3CL-protease (L89F) (B) protein. Detailed profiles of mobility (B-factors), eigenvalues, and deformability are shown.
