# Supplementary figures and images for: The Impact of Mutations on the Pathogenic and Antigenic Activity of SARS-CoV-2 during the First Wave of the COVID-19 Pandemic: A Comprehensive Immunoinformatics Analysis
Source: Vaccines (Basel). 2021 Nov 30;9(12):1410. doi: 10.3390/vaccines9121410 (PMC8705738; doi:10.3390/vaccines9121410)

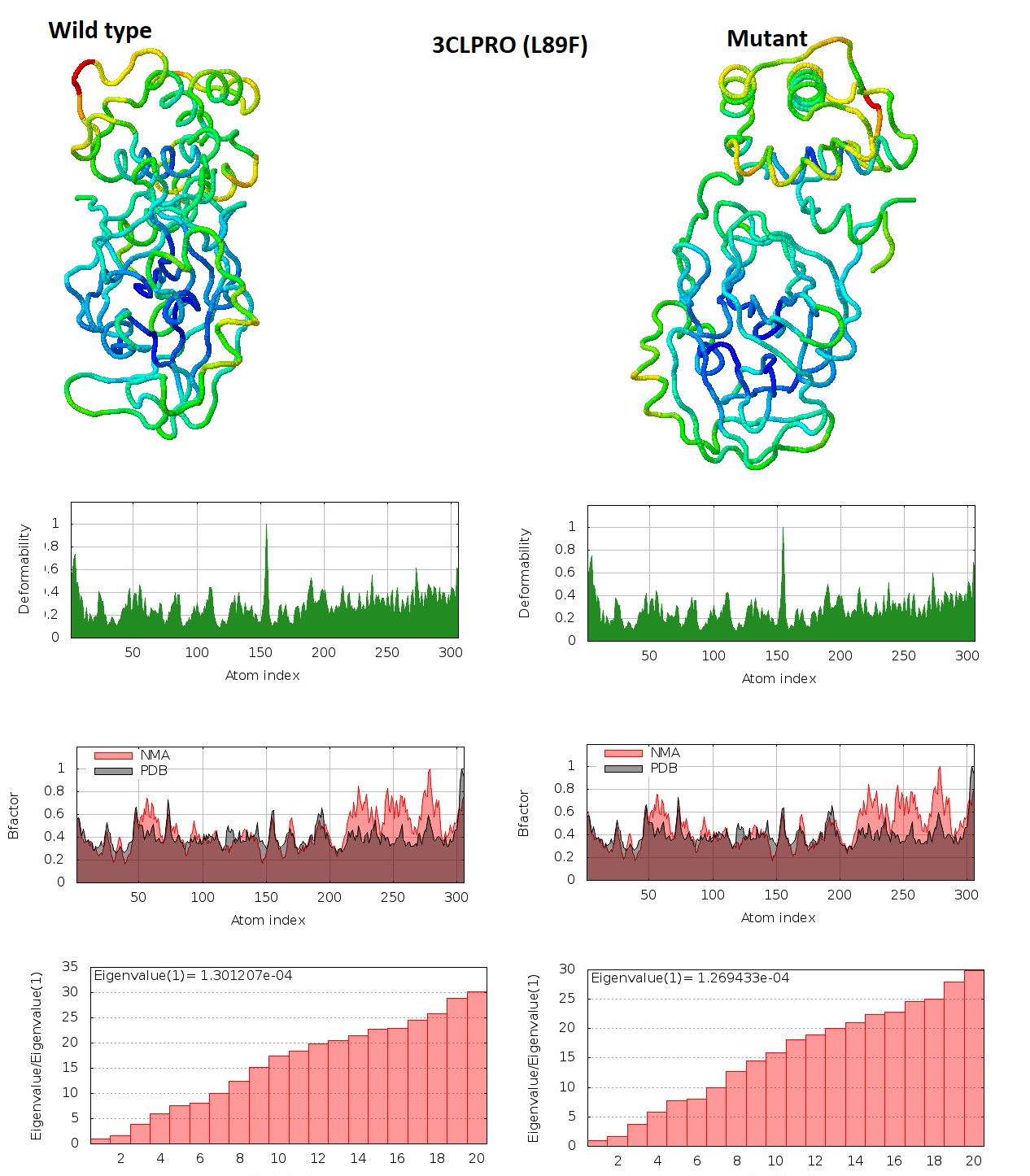

Supplement: Supplementary file 1 [file vaccines-09-01410-s001.zip › vaccines-1405579-supplementary.png]
